# CT-based measurement of visceral adipose tissue volume as a reliable tool for assessing metabolic risk factors in prediabetes across subtypes

**DOI:** 10.1038/s41598-023-45100-8

**Published:** 2023-10-20

**Authors:** Jihyun Kim, Keunyoung Kim

**Affiliations:** 1 Department of Nuclear Medicine and Biomedical Research Institute, Pusan National University Hospital, 179, Gudeok-Ro, Seo-Gu, Busan, Republic of Korea; 2 Department of Nuclear Medicine, College of Medicine, Pusan National University, Yangsan, 50612 Republic of Korea

**Keywords:** Endocrine system and metabolic diseases, Endocrinology, Endocrine system and metabolic diseases

## Abstract

Visceral adipose tissue (VAT) is a well-established risk factor for the development of diabetes in individuals with prediabetes. We aimed to evaluate various adiposity and metabolic indices, including visceral adipose tissue (VAT) volume measured by CT, in individuals with prediabetes, based on their different subtypes. This retrospective study included 78 prediabetes individuals aged ≥ 20 years whose VAT volumes were evaluated by CT. Individuals were classified into prediabetes subtypes: impaired fasting glucose (IFG), impaired glucose tolerance (IGT), and combined IFG and IGT (C-IFG/IGT). We conducted a comparison of the patients’ adiposity indices and their associations with one other, as well as with insulin resistance, based on the different prediabetes subtypes. Individuals with C-IFG/IGT had higher levels of visceral obesity than those with IFG or IGT. VAT volume was more strongly associated with insulin resistance than other adiposity indices, regardless of prediabetes subtypes. Additionally, VAT volume and liver attenuation index showed a significant correlation with the other indices studied across the prediabetes subtypes. CT-based measurement of VAT volume may be a valuable tool for evaluating metabolic risk factors among individuals with prediabetes.

## Introduction

Prediabetes, a high-risk factor for the development of type 2 diabetes mellitus (T2DM), is defined as a blood glucose level higher than normal but not yet at the diabetes diagnostic threshold, and is associated with an increased risk of cardiovascular disease (CVD)^1–3^. Visceral obesity is an important risk factor for CVD and T2DM, and the metabolic results of visceral obesity are associated with insulin resistnace^4–6^. Insulin resistance and a decline in pancreatic beta cell function are major pathophysiological factors for the development of diabetes in individuals with prediabetes^7^. The evaluation of visceral obesity may be a useful approach for predicting the risk of developing diabetes and CVD among individuals who have been diagnosed with prediabetes.

Based on the results of the oral glucose tolerance test (OGTT), prediabetes is classified as impaired fasting glucose (IFG), impaired glucose tolerance (IGT), or a combination of both (IFG and IGT). Different responses to the OGTT arise from differing pathophysiological mechanisms, including variations in the site of insulin resistance and insulin secretion patterns, among the different prediabetes subtypes^8^. Thus, the correlation between insulin resistance and visceral obesity may differ according to prediabetes subtype.

Anthropometric measurements such as body mass index (BMI), waist circumference (WC), and waist-to-hip ratio (W/H ratio) have been used in clinical settings to evaluate visceral obesity. However, given their indirect assessment of visceral adipose tissue (VAT) and racial/ethnic difference in the relationships between WC or BMI and VAT, these measurements have limitations^9^. Consequently, computed tomography (CT) is the gold standard for accurate quantitative evaluation of VAT^10^. VAT is an active endocrine organ that releases adipocytokines and is associated with inflammatory markers, hepatic steatosis, and metabolic risk factors, all of which may increase the risk of developing metabolic syndrome and T2DM^11–13^. Accurate measurement of VAT is therefore crucial for assessing the risk of diabetes and CVD among individuals with prediabetes.

In this study, we compared VAT volume measured by CT to other adiposity indices, in individuals with prediabetes. Furthermore, we evaluated the associations between adiposity indices and insulin resistance among individuals with prediabetes, based on the subtypes.

## Material and methods

### Study design and population

The electronic records of individuals with prediabetes at Pusan National University Hospital (Busan, Republic of Korea) were retrospectively reviewed between December 2017 and December 2019. Prediabetes was defined according to the American Diabetes Association criteria; therefore, we included those subjects with a fasting plasma glucose (IFG) level between 100–125 mg/dL or with a 2-h glucose level (IGT) between 140 and 199 mg/dL during a 75 g OGTT, and had a hemoglobin A1c (HbA1c) levels of 6.4% or below^1^. Individuals with prediabetes aged ≥ 20 years, who had abdominal CT screening for health check-up, suspected pancreatic disease, or follow-up imaging test for cancer, etc., were included in the study. Individuals were excluded from the study if they had undergone abdominal surgery or antidiabetic medications. The patients who are at the ongoing or progressive status of malignant disease, active fever, visible edema or active systemic comorbidity such as chronic renal disease, cirrhosis and heart failure which could significantly affect anthropometric and quantitative CT measurement were also excluded. Individuals with prediabetes were classified into subgroups according to prediabetes type (isolated IFG [I-IFG], isolated IGT [I-IGT], or combined IFG and IGT [C-IFG/IGT]). This study was conducted in accordance with the Declaration of Helsinki and approved by the Institutional Review Board named Pusan National University Hospital (IRB no. 2105-006-102, protocol code: 2021-NM-001). The requirement of written informed consent from enrolled subjects was waived by the Institutional Review Board of Pusan National University Hospital due to the retrospective study design.

### CT protocol and CT-based adiposity indices

**Figure 1:**
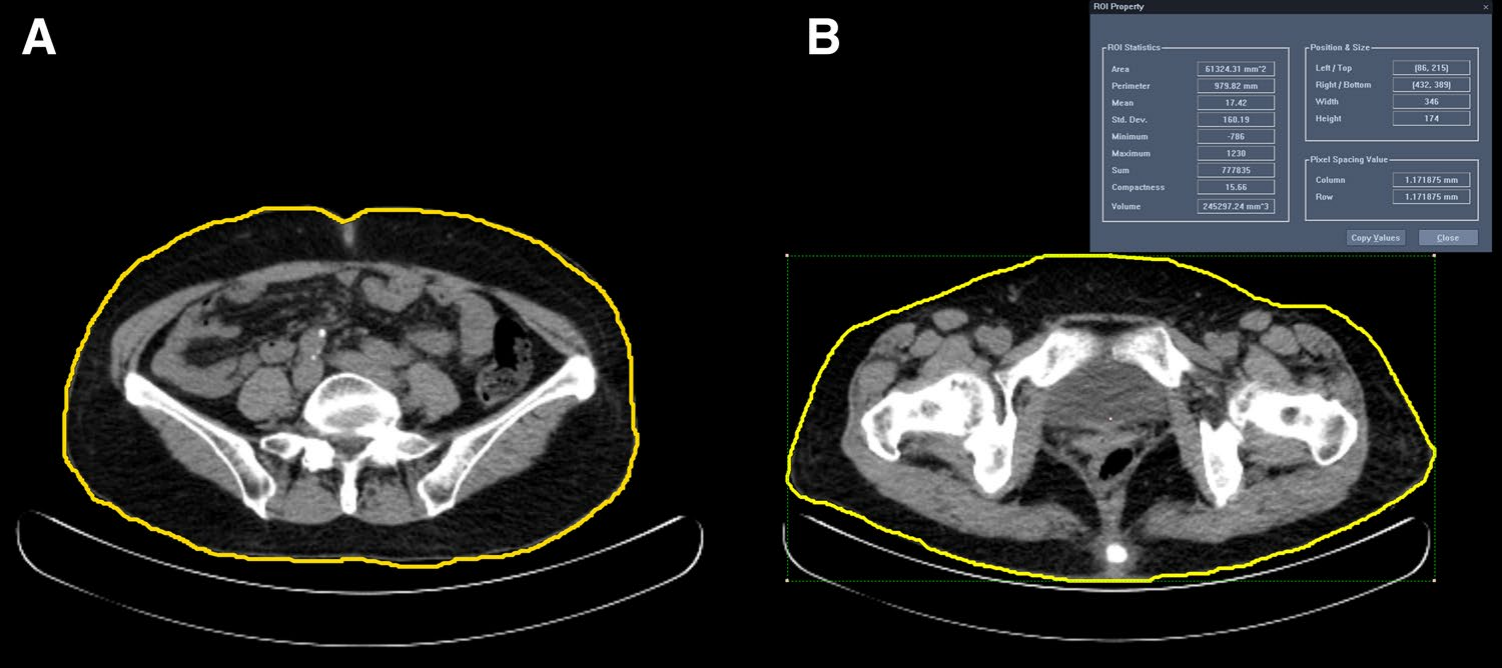
An example of region of interest (ROI)s on computed tomography. ROIs are used to obtain the waist circumference (WC) and hip circumference, and are manually drawn on the transaxial non-enhanced computed tomography image at the umbilicus level for WC (**A**) and the greater trochanter level of the femur (**B**).

Unenhanced spiral CT was performed using a Philips Brilliance 16-slice multidetector helical CT scanner (GEMINI TF CT; Philips, Eindhoven, Netherlands) at a voltage of 120 kVp, with a 3-mm slice thickness from the diaphragm to the mid-thigh. The VAT was manually defined as ranging from the diaphragm to the pelvic floor. The VAT volume was calculated by setting the attenuation values from -45 to -195 Hounsfield units (HU) using CT software (SIEMENS; Syngo CT basic evaluation). The WC and hip circumference (HC) were also measured using CT images. The perimeter of the free-drawn region of interest (ROI) at the umbilicus level was defined as the WC, and the ROI at the greater trochanter level of both femurs was defined as the HC (Fig. 1). The W/H ratio was calculated as the WC/HC ratio. The liver attenuation index (LAI) was calculated as follows: the average HU of five ROIs with 2–3 mm diameters within the liver was divided by the average HU of three ROIs within the spleen^14^.

### Laboratory assessments and metabolic indices

Height (cm) and weight (kg) were measured using an automatic height-weight scale (GL-150; G-tech, Seoul, Korea). Body mass index (BMI) was calculated as weight (kg) divided by height (m) squared.

Venous blood samples were collected from all participants in the morning after 12 h of overnight fasting. Biochemical assays were performed using an autoanalyzer (Hitachi 747; Hitachi Corp., Tokyo, Japan). Lipid profiles included total cholesterol (TC; mg/dL), triglycerides (TG; mg/dL), high-density lipoprotein (HDL) cholesterol (mg/dL), and low-density lipoprotein (LDL) cholesterol (mg/dL). Fasting plasma glucose (mg/dL) was measured by the glucose oxidase method using a Synchron LX20 (Beckman Coulter; Fullerton, CA, USA). Plasma insulin level (µU/mL) was determined using an enzyme immunoassay (Dainabot; Tokyo, Japan).

Insulin resistance was calculated using the homeostasis model – insulin resistance (HOMA-IR), by the following formula: fasting insulin (µU/mL) × plasma glucose (mg/dL)/405 and Matsuda insulin sensitivity index: calculated as 10,000/(fasting glucose (mg/dL) × fasting insulin (μU/mL) × mean glucose during OGTT (mg/dL) × mean insulin during OGTT (μU/mL))^0.5^. Insulin secretion was determined using the homeostasis model–beta-cell function (HOMA-beta), by the following formula: HOMA-beta = 360 × fasting insulin (μU/mL)/(fasting glucose [mg/Dl]—63)^15,16^.

The visceral adiposity index (VAI) was defined as follows: men: VAI = (WC/[39.68 + 1.88 × BMI]) × (TG /1.03) × (1.31/HDL); women: VAI = (WC/[36.58 + 1.89 × BMI]) × TG/ 0.81 × (1.52/HDL).

### Statistical analyses

All non-normally distributed variables are expressed as medians and interquartile ranges (IQRs; 25–75%). The Mann–Whitney U test was used to compare continuous variables between the two groups. Categorical group data were compared using the Chi-squared test. Spearman's rank correlation was applied to analyze the degree of association between the variables. To evaluate the relationship between adiposity or metabolic indices and HOMA-IR, we conducted a stepwise multiple linear regression by considering a set of potential variables. The set of variables includes BMI, WC, W/H ratio, LAI, VAI, VAT area and VAT volume, which were revealed as significant variables from the univariate analysis. Statistical analyses were performed using MedCalc® (version 16.4.3; MedCalc, Mariakerke, Belgium) and GraphPad Prism (ver 8.0; GraphPad Software, Inc., San Diego, CA, USA). Statistical significance was set at *p* < 0.05.

## Results

### Baseline characteristics and differences based on prediabetes subtypes

A total of 78 individuals diagnosed with prediabetes were enrolled (M:F = 14:64; median age, 60 years; IQR, 56–64 years). All study subjects were of Korean ethnicity. Among individuals with prediabetes, 32 (41.0%) had either I-IFG or I-IGT, while 46 (59.0%) had C-IFG/IGT. Approximately 30% (n = 24) of the individuals were treated for hypertension, and their systolic and diastolic blood pressures were relatively well controlled (Table 1).

**Table 1:** Baseline Individual Characteristics.

Number of participants	78
Phenotype of prediabetes	
Isolated IFG or IGT	32 (41.0%)
Combined IFG and IGT	46 (59.0%)
Sex	
Female	64 (82.05%)
Age (years)	60.0 [56.0;64.0]
Systolic blood pressure (mmHg)	122.5 [116.0;132.0]
Diastolic blood pressure (mmHg)	72.0 [67.0;79.0]
Medication for Hypertension	
Yes	24 (30.8%)
Administration of statin	
Yes	39 (50.0%)
HbA1c (%)	6.0 [5.9;6.2]
HOMA-IR	2.1 [1.7;3.1]
HOMA-beta	78.7 [51.9;106.9]
Matsuda insulin sensitivity index	4.0 [3.0;6.0]
Total cholesterol (mg/dL)	177.0 [154.8; 200.0]
Triglyceride (mg/dL)	116.0 [79.3;172.0]
LDL-cholesterol (mg/dL)	102.1 [85.5;121.0]
HDL-cholesterol (mg/dL)	54.0 [45.0;64.0]
Height (cm)	158.0 [153.0;163.4]
Weight (kg)	64.3 [56.0;70.0]
BMI (kg/m^2^)	24.9 [23.4;27.5]
WC (mm)	857.1 [810.5;909.4]
HC (mm)	912.4 [887.2;952.1]
WHR	0.92 [0.85;0.98]
VAT area (cm^2^)	46.6 [31.4: 62.6]
VAT volume (cm^3^)	3348.6 [2370.8;4180.9]
LAI	1.40 [1.23;1.55]
VAI	1.48 [0.99;2.63]

IFG: impaired fasting glucose, IGT: impaired glucose tolerance, HbA1c: hemoglobin A1c, HOMA: homeostasis model, IR: insulin resistance, LDL: low-density lipoprotein, HDL: high-density lipoprotein, BMI: body mass index, WC: waist circumference, HC: hip circumference, WHR: waist to hip ratio, VAT: visceral adipose fat tissue, LAI: liver-spleen-attenuation index, VAI: Visceral adiposity index.

Table 2 presents the individual characteristics based on subgroup analysis. Individuals with C-IFG/IGT showed significantly higher serum HbA1c levels and HOMA-IR values. Individuals with C-IFG/IGT shows a lower tendency in HOMA-beta and Matsuda insulin sensitivity index results compared to those with I-IFG or I-IGT, but the observed differences did not reach statistical significance. Compared to the individuals in the other prediabetes subtype groups, those with C-IFG/IGT had significantly higher WCs, W/H ratios, VAT areas, VAT volumes, and VAI values, as well as significantly lower LAI values.

**Table 2:** Comparison of Metabolic Characteristics according to Prediabetes Subtypes.

Diagnosis	Isolated IFG or IGT	Combined IFG and IGT	*p* value
	N = 32 (41.0%)	N = 46 (59.0%)	
Sex			0.826
Female	27 (80.0%)	37 (80.4%)	
Male	5 (20.0%)	9 (19.6%)	
Age (years)	55.0 [55.0;64.5]	62.0 [56.0;645.0]	0.446
Medication for Hypertension			0.866
Yes	10 (12.8%)	14 (18.0%)	
No	22 (28.2%)	32 (41.0%)	
Administration of statin			0.746
Yes	15 (19.2%)	24 (30.8%)	
No	16 (20.5%)	23 (29.5%)	
HbA1c (%)	6.00 [5.85;6.28]	6.05 [5.94;6.20]	< 0.001
HOMA-IR	1.80 [1.38;2.48]	2.51 [1.84;4.23]^a^	0.002
HOMA-beta	81.8 [52.2;81.8]	72.5 [51.3;105.2]	0.374
Matsuda insulin sensitivity index	5.2 [4.1–7.3]	4.1 [3.2–5.5]	< 0.001
Height (cm)	158.2 [153.3;163.5]	157.9 [153.0;162.0]	0.937
Weight (kg)	63.2 [56.5;67.4]	64.650 [55.0;75.0]	0.593
BMI (kg/m^2^)	24.7 [23.2;26.6]	26.2 [23.5;28.3]	0.204
WC (mm)	829.8 [772.2;857.8]	876.1 [830.8;940.6]^a^	0.001
HC(mm)	918.8 [883.3;951.1]	911.7 [887.2;959.0]	0.362
W/H ratio	0.87 [0.84;0.92]	0.96 [0.88;1.02]^a^	0.006
VAT area (cm^2^)	38.39 [27.14; 57.76]	54.60 [39.08; 64.90]^a^	0.006
VAT volume (cm^3^)	2649.3 [2015.9;3791.3]	3521.7 [2630.8;4405.3]^a^	0.020
LAI	1.43 [1.29; 1.60]	1.37 [1.19;1.52]	0.016
VAI	17.66 [12.70;22.72]	38.83 [21.64;64.91]^a^	< 0.001

IFG: impaired fasting glucose, IGT: Impaired glucose tolerance, HbA1c: hemoglobin A1c, HOMA-IR: insulin resistance homeostasis model, BMI: body mass index, WC: waist circumference, HC: hip circumference, W/H ratio: waist to hip ratio, VAT: visceral adipose fat tissue, LAI: liver-spleen-attenuation index, VAI: Visceral adiposity index.

### Multiple regression analysis between adiposity indices and insulin resistance

In our regression analysis between CT-based adiposity indices and insulin resistance, all adiposity indices were associated with HOMA-IR and Matsuda insulin sensitivity index in univariate analyses. However, multiple regression analyses showed that the W/H ratio (*β* = 0.307, *p* = 0.014) and VAT volume (*β* = 0.307, *p* = 0.010) remained independent predictors of HOMA-IR. For Matsuda insulin sensitivity index, only VAT volume (*β* = − 1.210, *p* = 0.004) was the only significant factor in individuals with prediabetes (Table 3).

**Table 3:** Multiple Regression Analysis among adiposity indicators, HOMA-IR and Matsuda insulin sensitivity index in prediabetes patients.

Variable	HOMA-IR				Matsuda insulin sensitivity index			
	Univariate analysis		Multivariate analysis		Univariate analysis		Multivariate analysis	
	*β*	*P*-value	*β*	*P*-value	*β*	*P*-value	*β*	*P*-value
BMI (kg/m^2^)	0.305	< 0.001			− 0.297	< 0.001		
WC (mm)	0.310	< 0.001			− 0.359	< 0.001		
W/H Ratio	0.305	< 0.001	0.307	0.014	− 0.288	< 0.001		
VAT area (cm^2^)	0.288	< 0.001			− 0.359	< 0.001		
VAT volume (mm^3^)	0.332	< 0.001	0.319	0.010	− 0.394	< 0.001	− 1.210	0.004
LAI	0.165	< 0.001			0.095	< 0.001		
VAI	0.237	< 0.001			− 0.040	< 0.001		

HOMA-IR: homeostasis model—insulin resistance, *β*: standardized regression coefficients, BMI: body mass index, WC: waist circumference, W/H Ratio: waist to hip ratio, VAT: visceral adipose tissue, LAI: liver-spleen-attenuation index, VAI: Visceral adiposity index.

### Heterogeneous associations in adiposity indicators according to prediabetes types

**Figure 2:**
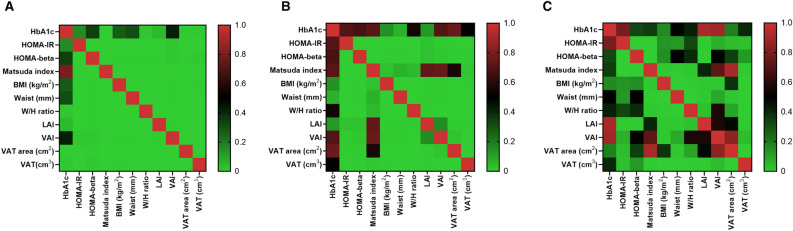
The heat-maps of colored by the *p* value-based significance of associations between the adiposity and metabolic indices. The lightest green color indicates *p* value of 0 and darkest red indicates *p* value of 1, which are presented as vertical shaped rectangle on the right side of the maps. Among the individuals with prediabetes, all adiposity and metabolic indices were significantly correlated with one another. Hemoglobin A1c showed the weakest correlation with the other metabolic parameters (**A**). Among individuals with isolated (I)-impaired fasting glucose (IFG) or I-impaired glucose tolerance (IGT), all adiposity and metabolic indices were significantly correlated with one another, except hemoglobin A1c (**B**). Among individuals with combined IFG/IGT, the associations between most of the adiposity and metabolic indices were insignificant except visceral adipose tissue volume and liver attenuation index (**C**).

In all individuals with prediabetes, we evaluated the correlations among all indices studied, including CT-based adiposity indices, HOMA-IR, and others. The weakest correlation was observed for serum HbA1c levels (Fig. 2A). During the subgroup analysis, significant associations were observed between the indices studied in individuals with I-IFG or I-GT (Fig. 2B). However, in individuals with C-IFG/IGT, the association between most of the indices studied were found to be insignificant and heterogeneous (Fig. 2C). Notably, VAT volume and LAI showed significant correlations with the other indices.

## Discussion

This study evaluated the relationships between various obesity or metabolic indices, such as VAT volume and insulin resistance, according to prediabetes subtypes. Despite showing no differences in BMIs, individuals with the C-IFG/IGT prediabetes subtype showed higher VAT volumes, VAT areas, WCs, and W/H ratios compared to those with the I-IFG or I-IGT subtypes. Moreover, a significant difference was observed in the visceral adiposity functional indices (VAT volume and LAI) between I-IFG or I-IGT and C-IFG/IGT. In this study, VAT volume demonstrated a better correlation with insulin resistance, a crucial factor in obesity-related comorbidities, compared to other obesity indices, across all prediabetes subtypes. These results were consistent with previous reports that showed that excess visceral fat was associated with prediabetes and T2DM, and that VAT volume was a stronger predictor for it than other anthropometric obesity indices^17–19^.

After stratifying for prediabetes subtypes according to I-IFG/I-IGT and C-IFG/IGT, the C-IFG/IGT group was found to exhibit significantly higher HOMA-IR and adiposity indices such as W/H ratio, VAT volume, and VAT area, when compared to the I-IFG and I-IGT groups. Prediabetes is a heterogeneous disease according to each patient’s response to the OGTT, and the pathophysiologic characteristics of IFG and IGT are considerably different in terms of insulin resistance and insulin secretion. IFG has a higher hepatic insulin resistance and reduced early phase (first 30 min) insulin response to oral glucose, whereas IGT has a higher muscle insulin resistance and reduced early and late phase (60–120 min) insulin secretion^20–22^. C-IFG/IGT have characteristics of both conditions, resulting in a higher risk of developing DM compared to I-IFG or I-IGT alone^8^. A previous study demonstrated that individuals with C-IFG/IGT had a significantly higher HOMA-IR compared to those with either I-IFG or I-IGT subtypes, whereas no significant differences were observed in BMI or W/H ratio^23^. In this study, individuals with C-IFG/IGT had significantly higher WCs, W/H ratios, and VAT volumes, compared to those with either the I-IFG or I-IGT subtypes. Previous studies have demonstrated variations in the association between anthropometric measures and risk factors in prediabetes, which is attributed to differences in measurement protocols across studies and ethnic variations. Specifically, the higher percentage of body fat observed in Asians at lower WC and BMI levels may contribute to these differences^24,25^. Notably, in this study, the correlations between most of the evaluated indices were diminished among individuals with C-IFG/IGT. However, CT-based measurement of VAT volume and LAI remained significantly associated with the others. Therefore, assessment of VAT volume and LAI may be valuable for evaluating metabolic risk in individuals with prediabetes, irrespective of their prediabetes subtypes.

CT-based VAT measurement is a potentially more precise and more objective method for VAT assessment than WC or W/H ratio but also has its limitations, including radiation exposure and high costs. However, volumetric quantification of VAT is highly reproducible, and there are significant differences between volumetric and single-slice measurements^26^. Abdominal CT scans have been frequently performed for health check-up, abnormal laboratory test suspected pancreatic disease in prediabetes, follow-up imaging test for cancer, and other medical conditions for the diagnosis and follow-up of diseases that affect abdominal organs in individuals with prediabetes. Several studies have already reported that the abdominal CT is useful for the patients with diabetes to assess the pancreatic tumor or pancreatic endocrine function^27–30^. Therefore, utilizing these abdominal CT images for VAT measurements can provide additional cardiometabolic information without additional radiation exposure as the enrolled participants in the current study could be evaluated for their CT-based VAT measurement using already acquired their abdominal CT.

In addition, NAFLD is highly prevalent in T2DM patients and is an early predictor of T2DM development^31^, as demonstrated by a recent meta-analysis that showed an increased risk of T2DM and metabolic syndrome in subjects with NAFLD^32^. Therefore, predisposed abdominal CT scans also provide information on non-alcoholic fatty liver disease (NAFLD)^33^. In the present study, there was no patient showed significant decrease in hepatic density suggesting hepatosteatosis. LAI results from abdominal CT scan showed an important difference among prediabetes subtypes, and was associated with insulin resistance and other adiposity indicators in individuals with C-IFG/IGT.

This study has some limitations. First, it was conducted retrospectively in a single medical center with a relatively small sample size (mainly women), especially in terms of the subgroup analysis. However, several previous reports have also shown that C-IFG/IGT had a higher correlation with central obesity and insulin resistance, agreeing with the results of our study^8,23^. Thus, the consistent correlation observed between VAT volume and insulin resistance across different subtypes of prediabetes indicates that VAT volume plays a crucial role in the development of DM in individuals with prediabetes. Second, there was a lower prevalence of I-IGT and an absence of normal glucose tolerance in this study population. However, our study demonstrated a significant difference in several clinical indices between individuals with a combined type of prediabetes subtypes and those with a single type of prediabetes subtypes. Further a large-population study that includes individuals with normal glucose tolerance would be needed in the future.

## Conclusion

There are differences in the significance of adiposity indices among the different subtypes of prediabetes. VAT volume has a stronger correlation with insulin resistance compared to the other obesity indices. Therefore, CT-based measurement of VAT volume may represent a valuable and reliable tool for evaluating metabolic risk factors among individuals with prediabetes.

## Data Availability

The datasets used and/or analyzed during the current study available from the corresponding author on reasonable request.
