# Author Correction: CT-based measurement of visceral adipose tissue volume as a reliable tool for assessing metabolic risk factors in prediabetes across subtypes

**DOI:** 10.1038/s41598-023-49371-z

**Published:** 2023-12-18

**Authors:** Jihyun Kim, Keunyoung Kim

**Affiliations:** 1 Department of Nuclear Medicine and Biomedical Research Institute, Pusan National University Hospital, 179, Gudeok-Ro, Seo-Gu, Busan, Republic of Korea; 2 Department of Nuclear Medicine, College of Medicine, Pusan National University, Yangsan, 50612 Republic of Korea

Correction to: *Scientific Reports* 10.1038/s41598-023-45100-8, published online 20 October 2023

The original version of this Article contained an error in the Acknowledgements section, where the grant number was incorrect.

“This research was supported by a Grant from the Korea Health Technology R&D Project through the Korea Health Industry Development Institute (KHIDI), funded by the Ministry of Health & Welfare, Republic of Korea (Grant Number: HI18C2383).”

now reads:

“This research was supported by a Grant from the Korea Health Technology R&D Project through the Korea Health Industry Development Institute (KHIDI), funded by the Ministry of Health & Welfare, Republic of Korea (Grant Number: HR20C0026).”

The original Article has been corrected.

